# Learning from clinical supervision – a qualitative study of undergraduate medical students’ experiences

**DOI:** 10.1080/10872981.2022.2048514

**Published:** 2022-03-06

**Authors:** Cathinka Thyness, Aslak Steinsbekk, Hilde Grimstad

**Affiliations:** Department of Public Health and Nursing, Norwegian University of Science and Technology (Ntnu), Trondheim, Norway

**Keywords:** Undergraduate medical education, psychological safety, clinical supervision, work-based learning, medical students

## Abstract

**Introduction:**

Clinical supervision is necessary to ensure students’ learning and patient safety. There is limited research on how medical students’ actions play into the dynamic of learning from clinical supervision. We aimed to explore undergraduate medical students’ experiences with learning from clinical supervision, focusing on students’ actions and interactions.

**Materials and methods:**

A qualitative study using semi-structured interviews with medical students at two English and four Norwegian universities. The main topics were students’ experiences with clinical supervision, what students’ felt helped them learn, and how they acted. Transcribed interviews were analysed thematically.

**Results:**

22 students participated. The actions participants described performing during supervision ranged from staying quiet to initiating active participation. They described that learning was more likely to take place when they took initiative, acted on opportunities to participate, and focused their attention on learning. When they did not feel safe, they were more likely to stay quietly in the background. When participants felt concerned for patients’ welfare their attention shifted away from learning. While if they were appropriately confident, they engaged in learning.

**Conclusion:**

Feelings of safety, patients’ being cared for, and confidence impacted on students’ actions and thus learning. Our findings suggest that when students feel psychologically safe, they are more likely to act and interact during clinical supervision. One way to improve psychological safety is to foster relationships between students and supervisors.

## Introduction

Supervision entails encounters between professionals that enables at least one of the participants to improve his/her performance, with the ultimate goal of improved quality of patient care and health outcomes [1–3]. Supervised clinical experience is an EU requirement for attaining a medical doctor degree [4].

In 2014, Dornan et al. presented a model of learning in clerkship [5], which they have continued to develop [6], based on a literature review. They described the clinical learning process as supported participation in practice and conclude that clinicians in the roles of mentors, preceptors, and supervisors aid student learning through providing affective, pedagogic and organisational support to students participating in practice [5,6]. However, students’ contribution was not mentioned [6] and the authors’ commented that there was little research on the interactional aspects of participation [5].

There are also few studies on clinical supervision of other health care students that mention students’ contribution and interactions. Pront, Gillham and Schuwirth reviewed what health care students perceive as good supervision in the sense that they learn from it [7]. They did a comprehensive search of major health and science databases to find articles on what helps students learn from supervision, and conducted a thematic analysis on the articles they identified. Apart from the joint work of establishing a relationship and developing common views on the purpose and process of supervision between the students and supervisors, their findings focused on the actions of the supervisor and did not address students’ actions and interactions [7].

Articles on students’ contribution in clinical learning have mostly looked at what characterises ‘good’ clinical medical students [8,9] or what is perceived as students’ roles [10–13]. Only Hauer et al [14]. and Nguyen et al [15]. explored students’ actions, both in the US. Hauer et al. reported on findings of how students act to change unsuccessful relationships [14], while Nguyen et al. reported on which students’ actions encouraged doctors to teach them [15].

Thus, there is some beginning interest in students’ actions during clinical learning, but there is a need for more studies on how students’ actions play into the dynamic of learning from clinical supervision. We therefore aimed to explore undergraduate medical students’ experiences with learning from clinical supervision, focusing on students’ actions and interactions.

## Materials and methods

### Study design

This was a qualitative study using individual semi-structured interviews with undergraduate medical students in Norway and England. A qualitative design was chosen because it allows for exploration of subjective experience [16] and can be particularly useful on topics where little is known from before [17]. Rather than seeking a true or false answer, qualitative research aims to understand social phenomena [16].

### Ethical approval

Approval was given by the Norwegian Centre for Research Data (reference number 904,206). For the English universities, permission was sought from the universities. Students were asked to sign the consent form before the interview and were informed orally and in writing that they could withdraw at any time without any explanation or repercussions.

### Participants

We aimed to include medical students of both genders, halfway through third year and above in five- or six-year programmes from at least four institutions and two countries to ensure variation in experiences.

To recruit participants, an e-mail, with a formal information letter and consent form attached, was distributed by staff or student representatives at the universities. To participate, students contacted the first author (CT) by e-mail. . Self-selected students were from all the relevant years and institutions, but due to underrepresentation of men, student representatives at two institutions were asked to encourage men to participate.

### Setting

Participants were recruited from 6 different universities. From one university they were purposefully sampled from the centralised, university hospital campus, and for two universities from district hospital campuses. The three remaining universities only had one, centralised campus. However, many of the participants had trained at both university and district hospitals and in general practice.

Participants were encouraged to talk about the situations that best illustrated their experiences with learning from clinical supervision. This could be in any speciality and health care setting as long as patients were involved. Both English and Norwegian medical students participate in practice individually and in groups.

English medical students have no license and cannot act independently. Norwegian medical students achieve a temporary license between half-way through 5^th^ and end of 5^th^ year. This allows them to work under a responsible supervisor and make prescriptions of non-addictive drugs.

### Data collection

We chose individual interviews to feasibly include students from a range of universities and year groups. The first author (CT) conducted interviews, four face-to-face and 18 by phone, between spring 2018 and spring 2020.

The interviews were semi-structured using an interview guide. The interview guide was based on literature on clinical teaching and learning identified through a PubMed search. The content was discussed with students involved in curriculum development at our institution. The structure of the guide and its sentences was discussed with qualitative researchers in a qualitative research course. During the study, the guide was revised so that it contained fewer, broader questions that asked for the same general content as the original questions in a less detailed manner and in a language adjusted to the students’ way of speaking. The topics included students’ concrete experiences with clinical supervision, what students’ felt helped them learn during and from supervision, and what they did during supervision. As long as the encounter with a supervisor pertained to patients, students were allowed to define what constituted supervision and which settings they talked about. The students were encouraged to speak freely, and interviews were continued until all topics were covered and participants felt like they had nothing more to add. Interviews lasted 30 to 90 minutes. Data collection and analysis took place concurrently. When new interviews supported identified themes rather than provided new ones, we concluded that sufficient saturation had been reached.

### Data analysis

The interviews were audio-recorded and transcribed verbatim while omitting all information that could identify individuals. We analysed the data following systematic text condensation [17,18], which is a descriptive, thematic cross-case analysis based on merging and synthesis of first-person descriptions of experiences. It consists of four iterative steps. Firstly, all authors read two-three transcripts to gain an overall impression and identify themes. We discussed our impressions and decided on preliminary themes. Secondly, CT used these themes to identify meaning units from the transcripts, and collections of meaning units were regularly discussed in meetings between the authors, where themes were modified along the way. In the third step, CT merged (‘condensed’) meaning units into a text expressing the content of participants’ utterances in first person. The authors went through several iterations of these three steps, including all authors reading more transcripts in detail before the final set of themes was decided upon. In the fourth step, CT with the critical input of HG and AS, used the condensed text to write a synthesis of the findings.

### Reflexivity

CT had obtained a medical degree from the Norwegian University of Science and Technology less than a year before commencing this study. However, she had no relationship with any of the participants. Nor had she any authority to affect their opportunities or progression in the medical school or beyond. Participants were informed that only CT would know their identity and that other researchers would have access to their interviews only as deidentified transcripts. HG is a medical doctor and professor in general medicine and behavioural medicine. In addition to research and teaching, she is involved in faculty development. AS is a professor in behavioural sciences in medicine and health service research. He has considerable experience in using and teaching qualitative methods, especially systematic text condensation. All three have been involved in medical education development locally and nationally. At the time of interviewing medical students from NTNU, AS was responsible for teaching and assessment of public health and alternative medicine in this medical school while HG had recently stepped down from her position as dean of medical education and had not resumed teaching medical students. As they would not know the identity of participants, their names lent legitimacy to the project, but they offered no potential benefits or repercussions to participants. The authors aimed to uphold reflexivity through CT keeping notes on her thoughts, experiences and reflections, and discussion among the authors for instance on how our emotional reactions to participants influenced our interpretations.

## Results

There were 22 undergraduate medical students from four Norwegian (N = 17) and two English (N = 5) universities who participated. The characteristics of the study programmes and participants are summarised in Tables 1 and 2

**Table 1.** Characteristics of included study programmes’ clinical teaching

	Duration of program (years)	Central or district	Longitudinal or block-based	Primary or specialist services	One or multiple encounters with supervisors
NCL[1]	5	District	Both	Both	Both
NTNU[2]	6	Both	Both	Both	Both
Oxon	6	Central	Block-based	Both	Both
UiB	6	Both	Block-based	Both	Both
UiO	6	Both	Both	Both	Both
UiT[3]	6	District	Both	Both	Both

**Table 2.** Characteristics of informants

**Age (years)**	
Mean	24,5
Range	20–32
**Gender (number)**	
Male	7
Female	15
**Year in medical school (number)**	
3^rd^	4
4^th^ in 6-year programme	4
Penultimate year	5
Final year	8

respectively. The number of recipients invited through automated e-mail lists were unknown.

NCL = Newcastle University, England; 1 Campus Northumbria; NTNU = Norwegian University of Science and Technology; 2 Campus Trondheim; Oxon = Oxford University, England; UiB = University of Bergen, Norway; UiO = University of Oslo, Norway; UiT = University of Tromsoe, The Arctic University of Norway; 3 Campus Finnmark

Participants described experiences from a large variety of contexts (e.g., speciality and geographical location including foreign exchanges), relationships (e.g., self-selected or mandated, one time encounter or multiple encounters) and level of supervision (from student only observing to student working alone).

The main findings were that participants described the best activities for learning during clinical supervision as those where they felt safe to participate to the best of their ability and receive corrections and feedback. Comfort, confidence and safety (in Norwegian ‘trygghet’) were commonly used words to describe favourable conditions for learning. The findings were categorised into the themes ‘Feeling safe with others’, ‘Distracted by concerns for the patients’, and ‘Students’ confidence’.

### Feeling safe with others

The participants described many situations where they did not feel safe with supervisors. Not feeling safe was often connected to a fear of being perceived as bothersome, stupid, incompetent or unprofessional if they said or did anything. When in unsafe situations, participants often chose to stay quietly in the background. To avoid unsafe situations, some participants said they tried to stick to the same department or doctor over time. Some left as soon as they could, either to find someone more approachable and positive to students or to spend the time reading. Some participants said that they started off with showing what they knew, which then allowed them to ask questions and make attempts when they were uncertain, without appearing stupid or incompetent.

‘I guess it’s about feeling safe in the situation you are in. That you feel that the person you’re asking don’t think you are mental for asking.’ (N6)

Participants’ perception of a safe atmosphere was said to mainly be based on interactions with supervisors and patients. For instance, patients saying they wanted to contribute to learning was felt to legitimise participants’ focusing on learning and taking more time asking questions and doing exams. Supervisors being welcoming, facilitating opportunities to participate, and exploring what participants felt comfortable doing made participants feel more at ease, able to cease opportunities and initiate further participation and interaction.

‘If they take more of an interest [in me as a student] early on, then I am more inclined to ask if I can take more responsibility.’ (N14)

Good relationships, especially with the supervisor, made participants less afraid of appearing stupid, thus making it easier to act on a wish to ask questions or participate. Participants build relationships by sticking to the same doctor over time and showing respect and interest. Some commented that they perceived supervisors felt safer to provide opportunities and feedback if they knew the student they were working with. Some participants also commented that positive relationships with peers who were present could provide support, which in turn made them feel safer to interact with supervisors and patients.

‘Especially if I’m with the same doctor for a whole day, or even two days … and we develop a good relationship, I learn a lot. Because we get to know one another, and especially if I’ve done all my little tricks of engaging early on, asking questions in a proper manner, making sure I behave in front of the patient and so on, then they trust me [… and] I get to try things, and also, they dare to correct everything I do that is not necessarily wrong, but that could have been done better.’ (N10)

### Distracted by concerns for the patients

Some of the participants’ stories revolved around whether the level of supervision was appropriate given the patient’s situation and the student’s competency. Participants new to the clinical environment or participants encountering something unknown to them wished for a supervisor to be present. Some would decline opportunities to interact with patients independently if they felt uncertain. If participants felt they interacted with patients without adequate support, they felt uncomfortable or abandoned which took their attention away from learning.

‘[S]he took me behind the curtain, where the patient lay, and said “here you go” and so I said “but, sorry, I haven’t done this before, could you tell me what to do?”. And then she didn’t say much, just “here you go” […] I felt it was very unethical, but I was already there, and she said: “put your fingers in there”, so I did, and then she left. So, then I stood there, trying to be professional.’ (N3)

Participants were also distracted if they thought the patient was uncomfortable or treated poorly during supervision. Examples of situations described as uncomfortable for the patient included many students in a room, the supervisor talking over the patient’s head, patients being treated in a way that objectified them or that the conversation was not related to the patient. Some participants tried to raise their voice or work around a doctor they disagreed with. Others described acting as the supervisor seemed to expect: Answering questions, acting on instructions or staying quiet. At the same time, participants registered the patients’ signs of discomfort.

‘I’ve experienced being […] expected to stand by the patient, who had just had surgery, and be quizzed […] we saw the patient getting paler and paler and almost sick and scared and shaky because they’re sitting there, listening to everything that can go wrong.’ (N1)

### Students’ confidence

Participants said confidence was important to whether students, both themselves and others, were active in and learned from clinical situations and that students differed in confidence. While some participants were confident enough to seek and grasp opportunities in any situation, others were more hesitant. Knowing the relevant questions to ask, what examinations to perform or having knowledge about the patients’ concern or condition made some participants more confident in participating on their own or supervisors’ initiative. Some expressed that feedback helped them build confidence. Their confidence was boosted when they had practiced a procedure or skill many times.

‘In the beginning it was scary […], as you get more comfortable in the role of a clinician, it doesn’t matter as much not knowing everything about the topic. Because, you know how to take the history, you know how to do the exam, and then it is okay.’ (N13)

Some said that as they advanced in medical school, they had come to realise that they needed to be more assertive to reach the required competence level, which made them more inclined to do so. Other participants said that having been provided with a checklist of things they should or had to do, had made them confident to ask for chances to practice. However, being dismissed when taking initiative had decreased participants’ confidence to ask. Some said they needed the supervisor to offer them opportunities if they were to do something they were not confident they mastered.

‘[I]f a doctor says to me “here comes a new patient, I want you to do the talking”, then that is something else, because when it comes from them, I feel that they know. They asked exactly because they know you’re not good at it, and they want you to have a go.’ (N8)

It was also commented that students could be too confident. This limited learning, as overly confident students could do independent consultations and loose the opportunity for corrective feedback based on direct observation.

## Discussion

The actions participants described performing during supervision ranged from staying quiet to initiating active participation. Participants described that focusing on learning, engaging with supervisors, taking initiative to take a history or do an exam, and asking questions helped them learn. Whether they felt safe with those present, how patients were treated and how confident they were affected their actions and thus learning.

Feeling safe or unsafe with supervisors, patients and peers was a re-occurring theme in participants stories of what affected their learning during clinical supervision. Participants often remained quiet out of fear of being in the way or appearing incompetent or stupid. When they felt safe, on the other hand, participants took initiative to participate and asked questions. Our participants’ descriptions of feeling safe with others bears resemblance with the concept of psychological safety. Psychological safety was initially conceptualised within the field of organisational psychology [19]. Kahn described psychological safety as ‘feeling able to show and employ one’s self without fear of negative consequences to self-image, status, or career’ [20]. Reviews on psychological safety seem to support our participants’ experience that their perception of safety mediates the effect of external factors on learning [21,22].

Participants described that knowing the supervisor or peers or having been properly introduced could foster feelings of safety. This is in line with findings from psychological safety research, where it has been found that positive leader relationships and peer support predicts psychological safety [21]. We also found that if it was clarified that participants were students and there to learn, they felt safer. This can be compared to role clarity, which has been found to be a predictor of psychological safety [21].

Participants commonly described positive interactions with supervisors or peers as a result of the structuring of placements or supervisors’ actions, but they also told stories of showing off what they knew to make an impression on supervisors that made them feel safe exposing what they did not know or mastered. Having the time to do this was described as one of the reasons why relationships of longer duration fostered better conditions for learning. Similarly, Hauer et al. has found that students in brief relationships struggled to share their knowledge and opinions and mostly react to supervisors, while students in longer relationships take part in a collaboration [14].

Based on our findings and previous research on psychological safety we can therefore suggest interventions by different groups to enhance learning behaviour through improving psychological safety. Students might improve psychological safety by building relationships with supervisors and supporting their peers. Educators can foster psychological safety through proper introductions, making roles explicit, facilitating students showing what they know, and building relationships. The programme leadership can structure the programme so that students have continuity with supervisors and peers. How to improve psychological safety can also be addressed in faculty development and in seminars preparing students for placements.

How patients were treated was described by participants to influence their learning through affecting whether they focused on the patient or learning. Participants both told stories of how supervisors making sure patients were well taken care of helped them focus on learning, and stories of how patients being treated poorly took their attention away from learning and made them worry about the patient. That students entering a helping profession are more concerned with patients well-being than their own learning is a good thing and can serve as an important reminder that patient welfare has to come first. It appears that teaching and learning activity that compromises the patient leads to distraction and discomfort rather than learning. This is a finding that can be used in faculty development to help educators feel that what is best for patients and students is aligned and that by taking care of the patient, they also take care of the student(s).

Lastly, we found that participants perceived that confidence affected their learning from clinical supervision through engagement in learning activity. Nguyen et al. also identified confidence as something that helps medical students learn in clerkships [15]. Participants in our study described confidence to be affected by previous experiences and thus possible to affect. Giving students the possibility of being prepared through clinical skills courses or letting them know what to have read up on in advance are suggestions for boosting students’ confidence and thus participation. Some participants mentioned that having check-lists of what to do made them feel legitimate asking to do those things. Check-lists could be a useful tool, but the cultural acceptability of such an approach is important to consider before transferring this finding.

A strength of this study is that it is from two countries, six universities and a range of clinical settings, making it more likely that the aspects identified are not a product of a specific setting, but of the meeting between supervisor, patient, and student. However, the study was conducted in two western cultures and the applicability of the findings in other settings must be considered by those familiar with those cultures. It is a limitation that most of the participants were self-selected. While we ensured variation in age, gender, year of study and training sites, participants might be similar in respects we did not pay attention to. For instance, participants were not asked about their socioeconomic or cultural background. A few mentioned being from another country than the one they studied in. However, they did not link this to how they learn from supervision. Had we enquired specifically or focused our analysis on differences in experiences between students from different sociocultural backgrounds, though, we might have identified ways in which these backgrounds shapes learning from supervision.

## Conclusions

Feelings of safety, patients’ being cared for, and confidence impacted on students’ actions and thus learning. Our findings suggest that when students feel psychologically safe, that patients are safe and that they are confident, they are more likely to act and interact during clinical supervision. Students, educators and programme leadership can all help foster psychological safety, for instance through facilitating relationships between students and supervisors.
